# Metabolite Profile Characterization of Cyanobacterial Strains with Bioactivity on Lipid Metabolism Using In Vivo and In Vitro Approaches

**DOI:** 10.3390/md21090498

**Published:** 2023-09-19

**Authors:** Tiago Ribeiro, Kristín Jónsdóttir, Rene Hernandez-Bautista, Natália Gonçalves Silva, Begoña Sánchez-Astráin, Afshin Samadi, Finnur F. Eiriksson, Margrét Thorsteinsdóttir, Siegfried Ussar, Ralph Urbatzka

**Affiliations:** 1 Interdisciplinary Centre of Marine and Environmental Research (CIIMAR/CIMAR), University of Porto, Avenida General Norton de Matos, s/n, 4450-208 Matosinhos, Portugal; tiago.amribeiro8@gmail.com (T.R.); nsilva@ciimar.up.pt (N.G.S.); sanastrainb@unican.es (B.S.-A.); 2 Faculty of Sciences, University of Porto, Rua do Campo Alegre, 1021, 4169-007 Porto, Portugal; 3 Faculty of Pharmaceutical Sciences, University of Iceland, Hofsvallagata 53, 107 Reykjavik, Iceland; kej@hi.is (K.J.); samadi.afshin84@yahoo.com (A.S.); finnure@hi.is (F.F.E.); margreth@hi.is (M.T.); 4 RG Adipocytes & Metabolism, Institute for Diabetes & Obesity, Helmholtz Diabetes Center, Helmholtz Munich, 85764 Neuherberg, Germany; renejavier.hernandez11@gmail.com (R.H.-B.); siegfried.ussar@helmholtz-munich.de (S.U.); 5 Joint Laboratory of Applied Ecotoxicology, Korea Institute of Science and Technology Europe (KIST EU), Campus E7.1, 66123 Saarbrucken, Germany; 6 ArcticMass, Sturlugata 8, 102 Reykjavik, Iceland; 7 German Center for Diabetes Research (DZD), 85764 Neuherberg, Germany

**Keywords:** cyanobacteria, metabolic disease, steatosis, thermogenesis, lipids, metabolite profile

## Abstract

Cyanobacteria have demonstrated their therapeutic potential for many human diseases. In this work, cyanobacterial extracts were screened for lipid reducing activity in zebrafish larvae and in fatty-acid-overloaded human hepatocytes, as well as for glucose uptake in human hepatocytes and *ucp1* mRNA induction in murine brown adipocytes. A total of 39 cyanobacteria strains were grown and their biomass fractionated, resulting in 117 chemical fractions. Reduction of neutral lipids in zebrafish larvae was observed for 12 fractions and in the human hepatocyte steatosis cell model for five fractions. The induction of *ucp1* expression in murine brown adipocytes was observed in six fractions, resulting in a total of 23 bioactive non-toxic fractions. All extracts were analyzed by untargeted UPLC-Q-TOF-MS mass spectrometry followed by multivariate statistical analysis to prioritize bioactive strains. The metabolite profiling led to the identification of two markers with lipid reducing activity in zebrafish larvae. Putative compound identification using mass spectrometry databases identified them as phosphatidic acid and aromatic polyketides derivatives—two compound classes, which were previously associated with effects on metabolic disorders. In summary, we have identified cyanobacterial strains with promising lipid reducing activity, whose bioactive compounds needs to be identified in the future.

## 1. Introduction

Nature is a very rich resource to discover new promising compounds serving as the basis for novel drug development projects with a focus on various diseases [1]. Some compounds from marine origin were already approved by the Food and Drug Administration (FDA) for the treatment of several diseases, and many are in clinical trials [2]. An important example is Brentuximab Vedotin, a compound that targets the CD30 protein, used for the treatment of Hodgkin’s lymphoma and anaplastic large-cell lymphoma [3]. This molecule was isolated from a mollusk, but is produced by symbiotic cyanobacteria, an important group of organisms, which have many compounds in different phases of clinical trials [2].

The success of cyanobacteria results from the multiplicity of biosynthetic pathways, their phylogenetic diversity, and their potential to produce diverse compounds [4]. The Blue Biotechnology and Ecotoxicology Culture Collection of Cyanobacteria (LEGE-CC) [5] is a source of new compounds that has shown promising results in different areas of research. In anticancer drug discovery, portoamides, a peptide mixture isolated from *Phormidium* sp., revealed cytotoxicity in 2D cell culture [6] and on the proliferative layer of multicellular spheroids from the cancer cell line HCT-116 [7]. A bioactivity screening with cancer cells grown as 3D spheroids identified seven strains with promising cytotoxicity [8]. Hierridin B was isolated from the marine cyanobacterium *Cyanobium* sp., targeting mitochondrial activity and function [9]. Nocuolin A, isolated from the cyanobacterial strain *Nodularia* sp., demonstrated potent cytotoxicity on colon cancer cells grown as 3D spheroids and inhibited mitochondrial oxidative phosphorylation [10].

Another attractive field for human applications with great pharmacological demand is obesity and its comorbidities. The incidence of obesity has risen worldwide [11], resulting in a rapid increase in the prevalence of related pathologies. Of major concern are: non-alcoholic fat liver disease (NAFLD), as a result of fat accumulation in the liver that can lead to cirrhosis and liver cancer [12]; type 2 diabetes, characterized by hyperglycemia due to insufficient insulin release from pancreatic beta cells [13]; and dyslipidemia, characterized by high blood levels of cholesterol and triglycerides or low levels of high-density lipoproteins [14]. The first evidence for the therapeutic potential of cyanobacterial compounds for the treatment of metabolic diseases was already demonstrated by previous studies, which identified lipid-reducing activities [15] and the inhibition of intestinal lipid absorption [16]. Known and novel chlorophyll derivatives were isolated successfully from cyanobacteria, which significantly reduced neutral lipid reservoirs in zebrafish larvae and in spheroids of differentiated murine preadipocytes [17].

The aim of this work was to screen a novel set of cyanobacteria from the Blue Biotechnology and Ecotoxicology Culture Collection (LEGE-CC) for their effect on the lipid metabolism, combining various in vivo (zebrafish larvae) and in vitro (cellular assays) approaches. After lab-scale growth, cyanobacterial biomass was extracted and fractionated, before being analyzed by various bioassays. The reduction of neutral lipids was evaluated in zebrafish larvae and in a steatosis model using human hepatocytes. Glucose uptake was assessed in human HepG2 cells, as well as the adipocyte differentiation and the induction of the mitochondrial uncoupling protein 1 in murine brown adipocytes. The metabolite profiling of the extracts by UPLC-Q-TOF-MS and a multivariate statistical approach were used to identify the most promising strains, with the potential to produce novel metabolites, as well as the metabolites, putatively involved in observed bioactivities. Cyanobacterial strains were discovered with novel bioactivities on lipid reduction in zebrafish and in fatty-acid-overloaded human hepatocytes, and on the increase of *ucp1* expression in brown adipocytes.

## 2. Results

### 2.1. In Vitro Analysis

#### 2.1.1. HepG2 Cells: Anti-Steatosis Activity, Glucose Activity, and Toxicity Evaluation

**Figure 1:**
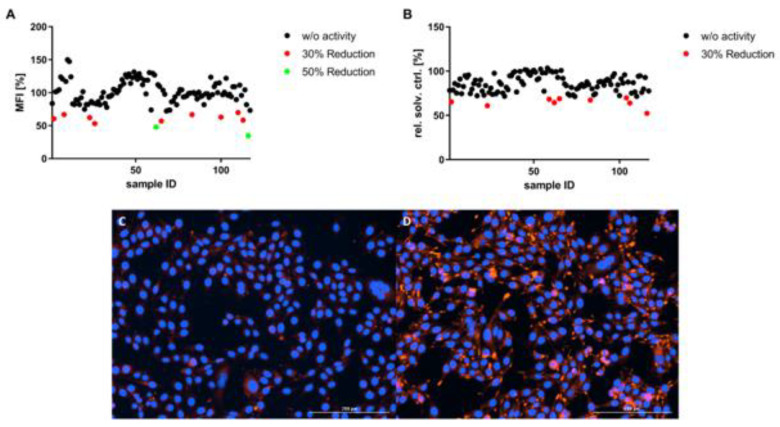
Bioactivity screening in a model of hepatic steatosis and toxicity evaluation. Data are presented as MFI relative to solvent control (0.5% DMSO + O62 μM) for (**A**) and percentage of viability relative to solvent control for (**B**). HepG2 cells were exposed for 6 h to 62 μM sodium oleate (O62 μM) and 10 μg/mL cyanobacterial extracts. The cytotoxicity of cyanobacterial extracts on HepG2 cells was evaluated after the anti-steatosis screening by the Sulforhodamine B assay. (**C**) shows a representative image of HepG2 cells without fatty-acid-overloading, whereas (**D**) shows the cells after being exposed to sodium oleate (blue color, DAPI staining, marks the nucleus, whereas orange marks the lipid droplets, by Nile red staining).

In the first set of experiments, fatty-acid-overloaded human HepG2 cells were used as an in vitro model for hepatosteatosis. These cells were used to evaluate the effects of cyanobacterial fractions on the lipid content. From the 117 screened fractions, 9 (7.7%) reduced the lipid content > 30% (mean fluorescence intensity of Nile red; MFI > 30%) and 2 (1.7%) reduced MFI > 50% (Figure 1A). Considering the toxicity results of the SRB assay, as described on Figure 1B, five fractions were selected as promising, which had bioactivity and no significant cytotoxicity (threshold of reduction of viability inferior to 30%). Those are fractions: 8, 26, 100, 110, and 113, obtained from five cyanobacterial strains (one marine and four freshwater strains); the identifications of strains are given in the Supplementary Table S1. Two fractions (10 and 11) showed an increase of the lipid content (~50%), but have not been further pursued in this study.

A separate set of experiments studying the effects of the cyanobacterial fractions on glucose uptake in HepG2 cells did not identify the bioactivity of any of the fractions (Supplementary Material, Figure S1).

#### 2.1.2. Brown Adipocyte and Differentiation and Thermogenic Expression

**Figure 2:**
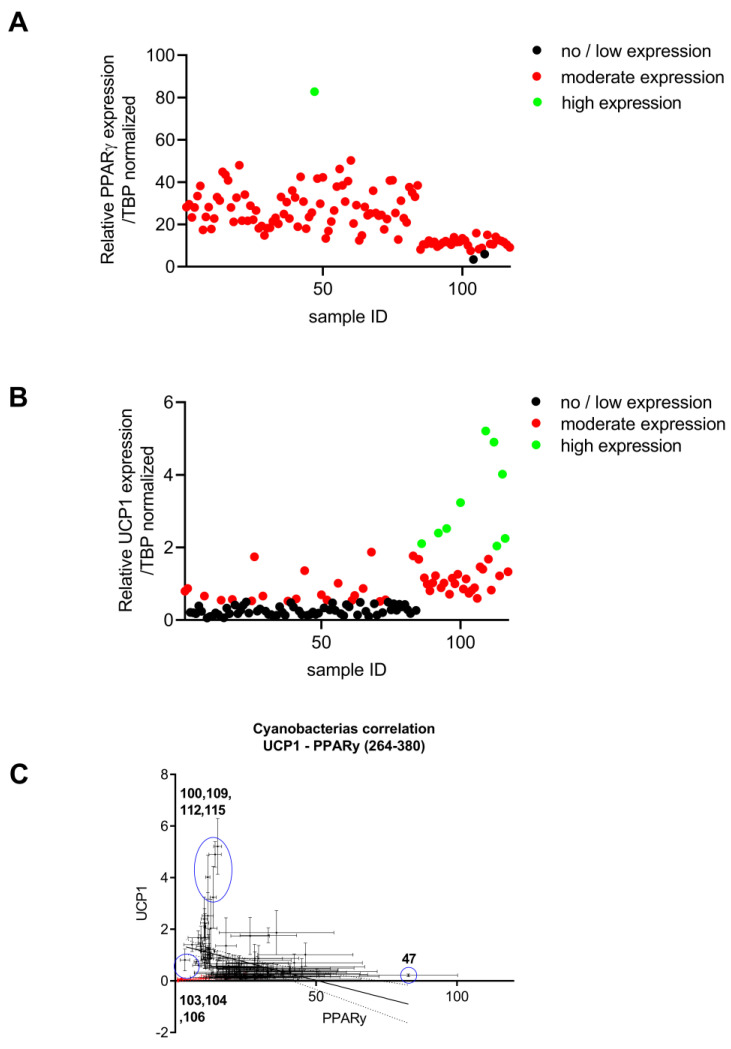
Analysis of mRNA expression of genes involved in (**A**) adipocyte differentiation (pparγ) and (**B**) thermogenesis (ucp1) by qPCR in brown adipocytes (n = 3). Values are shown as mean ± SEM. (**C**) Correlation between *ppary* and ucp1 mRNA expression identifies three different groups (low ucp1/low *ppary*; low ucp1/high *ppary*; high ucp1/high *ppary*).

In vitro differentiated murine brown adipocytes were used to test the activity of the cyanobacterial fractions on lipid storage and metabolism. These cells store, similar to white adipocytes, large amounts of energy in the form of triglyceride-filled lipid droplets. In addition, brown adipocytes also express the mitochondrial uncoupling protein 1 (*ucp1*), which is used to generate heat instead of ATP in the mitochondrial respiratory chain. Thus, monitoring the expression of *ucp1* can also serve as a marker for effects of the fractions thermogenesis. The cyanobacterial fractions were tested on their impact on adipocyte differentiation, as monitored by expression of the key adipogenic transcription factor *pparγ*. As shown in Figure 2, the fraction 47 significantly increased *pparγ* expression. *Ucp1* gene expression was significantly increased after exposure to fractions 92, 95, 100, 109, 112, and 115 (Figure 2B); the identifications of strains are given in the supplementary Table S1. The correlation analysis of *pparγ*/*ucp1* expression, as well as the dissociating effects of the fraction on adipogenesis versus mitochondrial function, revealed that fractions 26, 68, and 83 increased both *ucp1* and *pparγ* expression. Fraction 47 promoted the expression of *pparγ*, but inhibited *ucp1* expression. Fractions 103, 104, and 106 reduced both *ucp1* and *pparγ* expression, indicating a general inhibition of adipocyte differentiation or toxicity. Fractions 92, 95, 100, 109, 112, and 115 were selected as promising fractions, as they promoted ucp-1 expression beyond the levels that were expected based on the *pparγ* expression, suggesting a role in modulating mitochondrial function.

#### 2.1.3. In Vivo Analysis of Lipid Reducing Activity on Zebrafish Larvae

**Figure 3:**
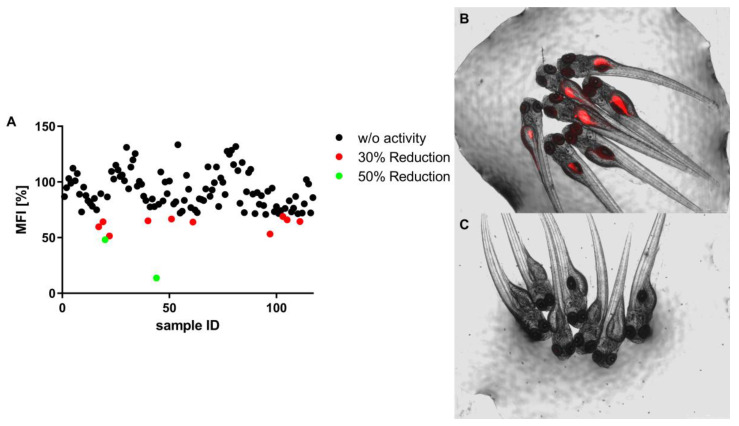
In vivo bioactivity screening using the zebrafish Nile red fat metabolism assay. (**A**) Data are presented as mean fluorescence intensity (MFI) relative to the solvent control. Zebrafish at 3 days post-fertilization (DPF) were exposed for 48 h to 10 μg/mL cyanobacterial extracts and lipids around the yolk sac and intestine were stained with Nile red. Representative images of zebrafish larvae (overlay of brightfield picture and red fluorescence channel). (**B**) Solvent control, 0.1% dimethyl sulfoxide (DMSO); (**C**) positive control, 50 μM REV. No cytotoxicity was observed.

The lipid-reducing activity from the cyanobacterial fractions was tested on zebrafish larvae using the Nile red fat metabolism assay. From the 117 screened fractions, 10 (8.5%) reduced the MFI > 30%, whereas 2 (1.7%) reduced MFI > 50%, as shown on Figure 3. The promising fractions (17, 19, 20, 22, 40, 44, 51, 61, 97, 103, 105, 111) belong to ten cyanobacterial strains, six from marine and four from freshwater ecosystems (see Supplementary Materials for strain identifications). No lethality or induction of malformations of the zebrafish larvae were observed for the exposure to any of the cyanobacterial fractions.

### 2.2. Chemical Analysis by Metabolic Profiling

Untargeted UPLC-Q-TOF-MS workflow was conducted to perform the metabolite profiling of 39 cyanobacteria strains. Each of the three different fraction types of cyanobacterial strains (fractions A, B, and C) were analyzed in separate batches. A total of 144 samples, including the quality control samples, were individually analyzed in triplicates. To ensure the accuracy and reproducibility of the UPLC-Q-TOF-MS analysis, pooled quality control (QC) samples were analyzed within each batch with the metabolic platform, and then data analysis was conducted with the principal component analysis (PCA) model to monitor system stability and data quality. The QC samples were injected 10 times at the start of each batch and then twice after every 11th sample. Each fraction did not show any indication of drifting over time and the pooled samples of each fraction clustered tightly when all pools were plotted together, indicating sustained analytical platform stability and precision throughout the runs. Multivariate data analysis (MVDA) was performed on the identified strains with substantially differing bioactivity compared to other strains within the same fraction. Different fraction types of cyanobacteria strains were analyzed separately, where one active strain was compared to non-active strains from the same fraction. Orthogonal projections to latent structures discriminant analysis (OPLS-DA) was applied to evaluate the difference between extracts with and without bioactivity, and to identify differential biomarkers, which have unique mass-to-charge values only present in the active fractions. Differential biomarkers were identified with VIP ≥ 1 from OPLS-DA and p(corr) > 0.05. The markers selected from the S-plots/loadings-plots were compared to the whole dataset in MarkerLynx and TargetLynx and explored for their presence in non-active strains. Each of the markers was a single mass peak, characterized by its specific retention time and accurate mass. The ellipse in the PCA plots stands for the 95% confidence interval.

**Figure 4:**
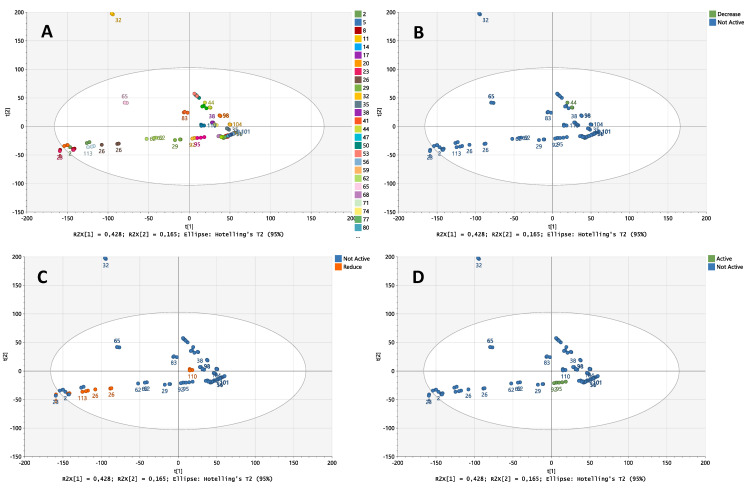
PCA score plots of fraction B. (**A**) Plot colored according to the different strains. (**B**) Plot colored according to the lipid reduction activity in zebrafish larvae. The green dots on the PCA plot represent fractions with decreased activity and the blue dots are non-active fractions. (**C**) Plot colored according to the anti-steatosis bioactivity. The orange dots represent fractions with decreased activity and the blue dots are non-active fractions. (**D**) Plot colored according to the ucp1 bioactivity. The green dots represent fractions with increased activity and the blue dots are non-active fractions.

Figure 4 shows the example of the PCA score plot of fraction B according to the strains and their bioactivities. In this fraction, the majority of the strains clustered together within the 95% confidence interval, except for three different strains coming from samples 8, 23, and 32.

This methodology was applied to fractions A, B, and C. In total, two markers were identified (see Table 1), which were different between zebrafish-lipid-reducing strains from fractions C and non-active fractions. One of them was uniquely found in the bioactive strain, whereas the other had higher peak intensities/areas in the active strain compared to the non-active strains. The remaining biomarkers derived from S-plot analyses were found to have similar peak area and peak height in non-active strains, as well as active strains. They were, therefore, not considered relevant mass peaks for the observed bioactivities.

Possible adducts for each identified mass were verified manually on the chromatograms, namely [M + H]^+^, [M + Na]^+^, [M + NH_4_]^+^, and [M + H − H_2_O]^+^ in positive ion mode. In the next step, the putative identification of the selected biomarkers was performed by searching in online databases (Metlin, Natural Product Atlas, and Dictionary of Natural Products) for an accurate molecular weight comparison and the identification of compounds (Table 2) using a mass error value of +/− 5 ppm. Drugs and toxicants were removed from the database search options. Two mass peaks were putatively identified as phosphatidic acid or aromatic polyketide derivatives. Further studies, such as MS/MS and NMR analysis, need to be carried out on these results to confirm the actual identity of the biomarkers responsible for the observed bioactivities.

## 3. Discussion

Cyanobacteria metabolites show very promising applications in the clinical field [18] as a result of high chemical diversity and biodiversity [19]. However, the majority of the strains remain unexplored for their bioactive potential. To evaluate the bioactive potential of new drugs, the combination of in vivo/in vitro models has proven to be very effective, namely by the combination of a fast screening on cell-based screenings with more complex animal models [20]. In this work, we applied a combination of cellular assays (steatosis model, adipocyte differentiation, 2-NBDG uptake), targeted screening (ucp1 mRNA expression), and in vivo screening in zebrafish larvae (Nile red fat metabolism assay). For all applied assays in this study, interesting bioactivity was observed in some of the cyanobacterial strains, which is in line with the previous identification of such bioactive potential in other cyanobacterial strains [15].

Metabolite profiling is a useful tool to select promising fractions based on their chemical profiles [21]. The great amount of data produced during the analysis of the metabolites leads to the need for a strong statistical and multidimensional analysis. The principal component analysis (PCA) leads to the identification of promising fractions, with different metabolites present on the bioactive samples. This is in agreement with the result of other studies using *Hapalosiphon* sp. and *Planktothricoides* sp., after changes on the light and temperature [22] or in the identification of novel stachybotrychromenes, from the fungus *Stachybotrys* sp. by LC-MS/MS [23]. In this work, metabolite profiling identified the most promising fractions between the active ones from the different assays. The fractions have the same main characteristics: (a) bioactivity towards metabolic disorders; (b) no toxicity, either in vitro or in vivo; (c) and the potential to produce novel metabolites. The combination of bioactivity screening with the metabolite profiling of active versus non-active strains led to the prioritization of two fractions, which reduced neutral lipid levels on zebrafish larvae on 5DPF embryos. Such bioactivity is reported for the first time for these strains. In our study, only two metabolite biomarkers were different in the bioactive strains, in contrast to [15], who observed many metabolite markers with associations to bioactivity. Putative identifications of those biomarkers revealed that they belonged to the compound classes of phosphatidic acid (PA) and aromatic polyketide derivatives. Fatty acid derivatives and their high bioactive potential for many human diseases can be seen in the literature [24]. Omega-3 polyunsaturated fatty acids (ω-3 PUFA’s), like eicosapentaenoic acid (EPA) and docosahexaenoic acid (DHA), are well studied fatty acids that are obtained from natural resources (vegetables and fish, for example), which reduced fat accumulation and improved metabolic diseases related with obesity (diabetes, cardiac diseases, and cholesterol levels) [25]. Derivatives of the phosphatidic acid are also identified as being involved in lipid metabolism, as cyclic molecules of PA are antagonists of PPAR-γ, a central molecule of adipogenesis [26]. Oxo-fatty acids also showed bioactivity towards PPAR receptors on both isoforms α/γ [27]. Future work is needed to identify the exact chemical structures of the involved PA and aromatic polyketide derivatives which showed bioactivity in this study. Several other natural products were already described to have beneficial effects on the reduction of lipid levels. Flavones, isolated from *Penthorum chinense*, a plant typical from some regions in Asia, had an anti-steatosis potential in a cellular model of steatosis (concentrations ranging from 100 µM to 10 µM) by inhibiting SIRT1/AMPK pathways [28]. Methanolic extracts of *Alisma orientalis*, a flowering plant, showed an inhibitory effect on lipid accumulation on an HepG2 steatosis model at a concentration relatively higher than the one used in this work (300 µg/mL), with the extract composed mainly of Alisol A, Alisol acetate, Alisol B, and Alisol B acetate [29]. The screening of a phenolic compound library revealed five promising compounds reducing the lipid levels in the zebrafish Nile red fat metabolism assay in a nanomolar range of concentrations [30]. Furthermore, new anthraquinones, questinol and citreorosein, isolated from a marine sponge-associated fungus, showed reduction on the lipid levels in the Nile Red assay, with an IC_50_ of 0.95 µM and 0.17 µM, respectively [31]. Additionally, microalgae extracts from *Chlorella vulgaris* at 10 µg/mL were shown to reduce the neutral lipid level in zebrafish larvae, particularly those from heterotrophic growth conditions; however, the responsible compounds were not yet identified [32].

The other assays (lipid reduction in fatty acid overloaded hepatocytes, glucose uptake in zebrafish larvae, and ucp1 mRNA expression in adipocytes) did not reveal promising metabolic markers after the combined analysis with the UPLC-Q-TOF-MS platform and multivariate statistics. Since no unique compounds were observed in these fractions, the quantities of responsible metabolites may be different in order to explain the observed bioactivities, which we will follow up in the future. In the hepatic model of steatosis, using fatty-acid-overloaded HepG2 cells, five strains revealed the potential in reducing lipid accumulation for the first time. Hepatic steatosis is a metabolic disorder related to obesity, leading to non-alcoholic fatty liver disease (NAFLD) due to high fat accumulation in the liver [33]. Several fractions affected the expression of both analyzed genes, pparγ and ucp1. PPAR-γ is a central regulator of lipid metabolism, stimulating adipocyte differentiation and the accumulation of lipids on the adipose tissue [34], whereas UCP1 is a key protein for thermogenesis, the regulation of energy consumption, and oxidative stress [35]. Based on the functions of these important regulators, the optimal condition would be a fraction that would increase UCP1, affecting energy consumption and the “burning” of fat reserves, while not affecting or even reducing PPARγ, used as a adipocyte differentiation marker in the brown adipocytes. In this work, a total of six fractions increased ucp1 mRNA expression. Interestingly, just one fraction showed promising activity on two different assays (fraction 100, reduction of lipid accumulation on HepG2 cells and increase on ucp1 levels), whereas all the others were active in only one of the tested assays. It should be expected that active fractions could be active in more than one assay (like in both lipid reduction assays), but results may differ due to adsorption, distribution, metabolization, or excretion [36] in the chosen in vivo and in vitro approaches.

## 4. Conclusions

Thirty-nine cyanobacterial strains were grown and a library of 117 chemical fractions was created. Twenty-three of them revealed promising bioactivity on metabolic disorders, especially on lipid reduction on zebrafish embryos, a reduction of steatosis on a cell model, and an increase of ucp1 mRNA expression. For lipid reduction in zebrafish larvae, two metabolic markers were identified in two strains, which were not present in non-active strains. These markers can be related to compound classes, which are known for their biological effects on lipid metabolism, such as phosphatidic acid and aromatic polyketide derivatives. Future work needs to elucidate the chemical structure of those compounds and their biological mode of action.

## 5. Materials and Methods

### 5.1. Cyanobacterial Growth and Screening Library Elaboration

A total of 39 Cyanobacteria strains were selected from Blue Biotechnology and Ecotoxicology Culture Collection (LEGE-CC) [5] and are listed in Supplementary Table S1. Culture conditions, biomass harvesting, and the subsequent fractionation by increased polarity extraction are described in [15]. The fractions obtained (3 for each strain, 117 in total) were then applied on the different bioassays. Fraction A corresponds to extraction with Hexane (Hex), Fraction B extraction with Ethyl-Acetate (EtOH), and Fraction C extraction with Methanol (MeOH).

### 5.2. Bioactivity Screening on HepG2 Cell Line and Toxicity Evaluation

HepG2 cells from the American Type Culture Collection (ATCC) (Manassas, VA, USA) were used for the anti-steatosis and glucose uptake assays. Cells were cultured in Dulbecco Modified Eagle Medium (DMEM) (Gibco, Thermo Fischer Scientific, Waltham, MA, USA) supplemented with 10% (*v/v*) fetal bovine serum (FBS) (Biochrom, Berlin, Germany), 1% penicillin/streptomycin (100 IU/mL and 10 mg/mL, respectively) (Biochrom), and 0.1% amphotericin (GE Healthcare, Little Chalfont, UK). Both bioassays and cytotoxicity evaluation were performed as described in [15]. Briefly, for the anti-steatosis, screening cells were seeded at 104 cells/well and, after 24 h, the medium was changed to DMEM without FBS supplemented with 62 μM sodium oleate (Sigma-Aldrich, St. Louis, MO, USA) and exposed to fractions and controls. After 6 h, 75 ηg/mL Nile red (Sigma-Aldrich) and 10 μg/mL Hoechst 33342 (HO-33342) (Sigma-Aldrich) were added and fluorescence was read with a Synergy HT Multi-detection microplate reader (Biotek, Bad Friedrichshall, Germany) at 485/572 nm excitation/emission for Nile Red and 360/460 nm for HO-33342. After the assay, the cells were fixed and a sulforhodamide B (SRB) (MP Biomedicals, LLC, Illkirch-Graffenstaden, France) assay was performed to access cytotoxicity. For the glucose uptake assay, cells were seeded at 105 cells/well and, after 24 h, placed under starvation in Hanks Buffered Salt Solution (HBSS) (0.137 M NaCl, 5.4 mM KCl, 0.25 mM Na_2_HPO_4_, 0.44 mM KH_2_PO_4_, 1.3 mM CaCl_2_, 1.0 mM MgSO_4_, 4.2 mM NaHCO_3_, glucose free) for 16 h. Then cells were exposed to the fractions and controls for 2 h, followed by 1 h with 100 μM 2-NBDG. The cells were washed and the fluorescence was read at 485/535 nm excitation/emission at Fluoroskan Ascent CF (MTX Lab Systems, Bradenton, FL, USA). The cytotoxicity was analyzed by an MTT assay. All the screenings were performed in two independent assays in triplicates per sample.

### 5.3. pparγ and ucp1 mRNA Expression on Differentiated Adipocytes by Real-Time PCR

For mRNA expression analyses, RNA from differentiated brown adipocytes was isolated using the QuickExtractTM RNA extraction kit (Epicentre Biotechnologies, Madion, WI, USA), following the manufacturer’s instructions. The synthesis of cDNA was performed in a Thermo Cycler by using a high-Capacity cDNA reverse transcription kit (Applied Biosystem, Foster City, CA, USA) according to the manufacturer protocol. Real-time PCR with SYBR green was performed using iTaqTM Universal SYBR^®^ Green Supermix (BIO-RAD, Hercules, CA, USA) in a CFX384 Touch Real-Time PCR Detection System (BIO-RAD, Hercules, CA, USA). Relative mRNA expression was calculated after normalization by TATA-binding protein (Tbp) expression. Primer sequences are listed in the Supplemental Table S2. Differential expression levels were calculated via the ΔΔct method [37].

### 5.4. Nile Red Fat Metabolism Assay on Zebrafish Larvae

The lipid reduction on zebrafish larvae was performed by a Nile red fat metabolism assay, as described in [15]. Briefly, 3-day post fertilization (DPF) larvae, raised in egg water (60 µg/mL marine salt) with 200 µM 1-phenyl-2-thiourea (PTU), were exposed to the test cyanobacterial fractions at a concentration of 10 µg/mL. Fractions and water were renewed daily until 5 DPF and DMSO (0.1%) and resveratrol (REV, at 50 µM) were used as solvent and positive controls, respectively. After overnight staining with Nile red at 10 ng/mL, larvae were anesthetized and imaging was performed with a fluorescence microscope (Olympus BX-41, Hamburg, Germany). Fluorescence intensity was quantified on each individual zebrafish larvae by ImageJ [38].

### 5.5. Chemical Analysis by Metabolite Profiling

The metabolic profiling of cyanobacterial extracts was performed using a Waters ACQUITY UPLC system (Waters, Milford, MA, USA), coupled to a Waters Synapt G1 mass spectrometer equipped with an electrospray ionization (ESI) probe (Waters, Wilmslow, UK). Chromatographic separation was carried out on a reverse-phase analytical column ACQUITY UPLC BEH C18 (2.1 × 100 mm, 1.7 µm) (Waters, Milford, MA, USA) at 60 °C. The mobile phases used for elution were A: water containing 0.1% formic acid (Sigma-Aldrich) and B: acetonitrile (HPLC grade, Merck, Germany) containing 0.1% formic acid. The flow rate was set at 0.45 mL/min and the injection volume was 3 µL. A linear gradient was used, starting at 65% B going to 100% in 8 min, followed by a column clean up at 100% B for 0.5 min and reconditioning at the initial conditions for 1.5 min. The total chromatographic run time was 12 min. The sample manager was maintained at 10 °C. Mass spectral detection was carried out in positive (ESI+) ion acquisition mode. The conditions of the ESI source were set as follows: capillary voltage, 3.2 kV; cone voltage, 42 V; source temperature, 125 °C; desolvation temperature, 450 °C; gas flow rate of 800 L/h (N_2_); cone gas flow rate 50 L/h. Leucine enkephalin was used as a Lockspray reference. All the fractions were run at the same time in separate batches for each fraction. The mass data were collected at the range of *m*/*z* 100–1500. The acquisition was controlled by MassLynx software 4.2 (Waters, Milford, MA, USA).

### 5.6. Statistical Analysis

The raw data files obtained from UPLC-Q-TOF-MS runs were analyzed using MassLynx v4.2, MarkerLynx v4.2, and Targetlynx v4.2 software (Waters). The extracted ion chromatograms were visualized using MarkerLynx. Markers at the range of 150 and 1500 Da were analyzed with an intensity threshold of 200 to 350 counts and with retention time and mass windows of 0.20 min and 0.050 Da, respectively. The noise level was set to 5.00. The replicate minimum was set to 50% for fractions B and C. The starting time interval was 0.8 min for fraction A and 0.4 min for fractions B and C. The end-time interval of the chromatograph was set to 11.20 for fractions A and B and 11.10 for fraction C. The variables in the dataset generated by Markerlynx were verified using Targetlynx software. The TargetLynx peak values were added to the dataset and the data were then normalized to total peak intensity before importing into SIMCA v17.0 (Umetrics, Umea, Sweden). Pareto scaling was applied before multivariate data analyses, including PCA and OPLS-DA, were performed.

### 5.7. Metabolite Online Search

The identified biomarkers masses were searched on online databases of chemical compounds such as METLIN [39], Natural Product Atlas [40], and Dictionary of Natural Products (https://dmnp.chemnetbase.com/chemical/ChemicalSearch.xhtml?dswid=5891, accessed on 11/10/2022). Masses were searched on a limit range of 5 ppm from the biomarker mass without the mass of the correspondent adduct (+H^+^, +Na^+^, +K^+^, +H-H_2_O^+^).

## Figures and Tables

**Table 1:** Summary of promising fractions with relevant bioactivities towards obesity, steatosis, and mRNA gene expression (thermal energy release).

Bioactivity	Selected Fraction/Sample No	Number of Detected Biomarkers
Zebrafish lipid reduction	LEGE07459C/#105 LEGE06114C/#51	1 1

**Table 2:** Possible identifications from METLIN, Natural Product Atlas (NPA), and Dictionary of Natural Products (DNP) databases of the potential metabolic markers from bioactive fractions, with mass error (+/− 5 ppm) and proposed structures for the molecular formulas. Zf-NR, zebrafish Nile red assay.

Retention Time (min)	Mass-to-Charge Ratio (*m*/*z*)	Strain	Bioactivity	Peak Area	Adduct Molecular Weight (MW)	Annotated Molecular Formula (METLIN)	Mass Error (ppm) (METLIN)	METLIN Proposed Structure
7.69	723.4933	LEGE 07459C	Zf-NR	34	[M + H]^+^, 723.4935	C39H72NaO8P	0	Dioleoyl phosphatidic acid
					[M + H]^+^, 723.4959	C41H71O8P	3	PA(16:1(9Z)/22:4(7Z,10Z,13Z,16Z)) PA(18:2(9Z,12Z)/20:3(8Z,11Z,14Z) PA(18:3(6Z,9Z,12Z)/20:2(11Z,14Z) PA(18:3(9Z,12Z,15Z)/20:2(11Z,14Z)) and 10 other proposed structures
6.44	525.4117	LEGE 06114C	Zf-NR	113	[M + H]^+^, 525.4116	C31H51N5O2	0	N-[10-(4-{(E)-[4 (Octyloxy)phenyl]diazenyl}phenoxy) decyl] methanetriamine 1-Naphthaleneacetonitrile, alpha,alpha-bis(2-(bis(3-methyl-2-butenyl)amino)ethyl)-

## Data Availability

Data is contained within the article or Supplementary Material.

## Supplementary Materials

The following supporting information can be downloaded at: https://www.mdpi.com/article/10.3390/md21090498/s1, Table S1: Library of Screening Fractions; Table S2: List of qPCR primers used in bioactivity screening; Figure S1: Bioactivity screening on the glucose uptake using HepG2 cell line (top) and bioactivity confirmation of the positive hits (bottom); Figure S2: S-plot for fraction C, highlighting two potential biomarkers from fraction C in regard to Zf-NR bioactivity.
