# Supplementary material for: Metabolite Profile Characterization of Cyanobacterial Strains with Bioactivity on Lipid Metabolism Using In Vivo and In Vitro Approaches
Source: Mar Drugs. 2023 Sep 19;21(9):498. doi: 10.3390/md21090498 (PMC10533020; doi:10.3390/md21090498)
Supplement: Supplementary file 1 [file marinedrugs-21-00498-s001.zip › marinedrugs-2592505-supplementary.pdf]

Supplementary Material

Table S1: Library of Screening Fractions. Each strain was extracted using three different solvents (A-Hexane; B-Ethyl Acetate; C-Methanol), creating three chemical fractions per strain. All the fractions were stored in DMSO at a concentration of 10 mg/mL.

LEGE CODE	Strain	Origin	Chemical Fraction	Library Number
LEGE 06077	*Nostoc cf. edaphicum*	Fresh Water	A	1
			B	2
			C	3
LEGE 07299	*Nostoc sp.*	Fresh Water	A	4
			B	5
			C	6
LEGE 06158	*Nostoc sp.*	Marine	A	7
			B	8
			C	9
LEGE 07365	*Nostoc sp.*	Fresh Water	A	10
			B	11
			C	12
LEGE 13413	*Nostoc sp.*	Marine	A	13
			B	14
			C	15
LEGE 03282	*Cuspidothrix issatschenkoi*	Fresh Water	A	16
			B	17
			C	18
LEGE 03285	*Cuspidothrix issatschenkoi*	Fresh Water	A	19
			B	20
			C	21
LEGE 00234	*Dolichospermum sp.*	Fresh Water	A	22
			B	23
			C	24
LEGE 04288	*Nodularia sp.*	Fresh Water	A	25
			B	26
			C	27
LEGE 06071	*Nodularia sp.*	Marine	A	28
			B	29
			C	30
LEGE 06188	*Calenema singularis*	Marine	A	31
			B	32
			C	33
LEGE 06123	*Gloeocapsopsis crepidinum*	Marine	A	34
			B	35
			C	36
LEGE 13421	*Gloeocapsopsis sp.*	Marine	A	37
			B	38
			C	39

LEGE 06111	*Calothrix sp.*	Marine	A<br>B<br>C	40<br>41<br>42
LEGE 06122	*Calothrix sp.*	Marine	A<br>B<br>C	43<br>44<br>45
LEGE 06105	*Plectonema cf. radiosum*	Marine	A<br>B<br>C	46<br>47<br>48
LEGE 06114	*Plectonema cf. radiosum*	Marine	A<br>B<br>C	49<br>50<br>51
LEGE 07159	*Rivularia sp.*	Marine	A<br>B<br>C	52<br>53<br>54
LEGE 06147	*unidentified Pleurocapsales*	Marine	A<br>B<br>C	55<br>56<br>57
LEGE 10410	*unidentified Pleurocapsales*	Marine	A<br>B<br>C	58<br>59<br>60
LEGE 11391	*Geitlerinema sp.*	Marine	A<br>B<br>C	61<br>62<br>63
LEGE 11393	*Geitlerinema sp.*	Marine	A<br>B<br>C	64<br>65<br>66
LEGE 07176	*Jaaginema litorale*	Marine	A<br>B<br>C	67<br>68<br>69
LEGE 06100	*Lusitaniella coriacea*	Marine	A<br>B<br>C	70<br>71<br>72
LEGE 06101	*Nodosilinea nodulosa*	Marine	A<br>B<br>C	73<br>74<br>75
LEGE 07164	*Schizothrix aff. septentrionalis*	Marine	A<br>B<br>C	76<br>77<br>78
LEGE 06141	*cf. Oculatella sp.*	Marine	A<br>B<br>C	79<br>80<br>81
LEGE 10370	*cf. Phormidesmis sp.*	Marine	A<br>B<br>C	82<br>83<br>84
LEGE 06003	*cf. Romeria sp.*	Marine	A<br>B<br>C	85<br>86<br>87
LEGE 06208	*Tychonema sp.*	Marine	A<br>B<br>C	88<br>89<br>90

LEGE 00036	*Synechocystis salina*	Marine	A<br>B<br>C	91<br>92<br>93
LEGE 00037	*Synechocystis salina*	Marine	A<br>B<br>C	94<br>95<br>96
LEGE 06106	unidentified *Nostocales*	Marine	A<br>B<br>C	97<br>98<br>99
LEGE 12447	*Geminobacterium atlanticum*	Fresh Water	A<br>B<br>C	100<br>101<br>102
LEGE 07459	*Myxosarcina sp.*	Marine	A<br>B<br>C	103<br>104<br>105
LEGE 06146	*Nostoc sp.*	Marine	A<br>B<br>C	106<br>107<br>108
LEGE 03284	*Cuspidothrix issatschenkoi*	Fresh Water	A<br>B<br>C	109<br>110<br>111
LEGE 00248	*Dolichospermum sp.*	Fresh Water	A<br>B<br>C	112<br>113<br>114
LEGE 00263	*Dolichospermum sp.*	Fresh Water	A<br>B<br>C	115<br>116<br>117

Table S2: List of qPCR primers used in bioactivity screening.

Gene	Forward primer (5´-3´)	Reverse primer (5´-3´)
UCP1	AGCCATCTGCATGGGATCAAA	GGGTCGTCCCTTTCCAAAGTG
PPARγ	CCCTGGCAAAGCATTTGTAT	GAAACTGGCACCCTTGAAAA
TBP	ACCCTTCACCAATGACTCCTATG	TGACTGCAGCAAATCGCTTGG

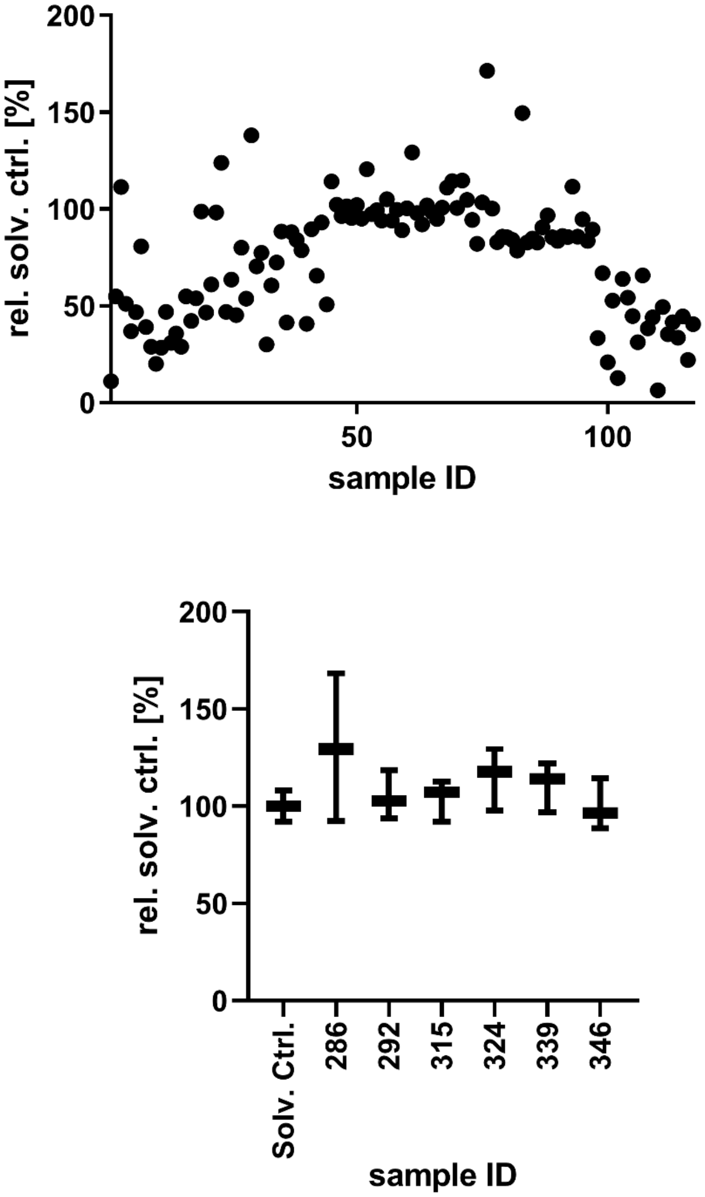

Figure S1: Bioactivity Screening, on the glucose uptake, using HepG2 cell line (top) and bioactivity confirmation of the positive hits (bottom). Data is presented as MFI relative to solvent control (0.5% DMSO) for both graphs. Briefly, 24 hours after seeding, cells were placed under starvation in HBSS for 16 hours. Afterwards, cells were exposed to the fractions and controls for 2 hours followed by 1 hour with 100 μM 2-NBDG. Cells were washed and fluorescence read.

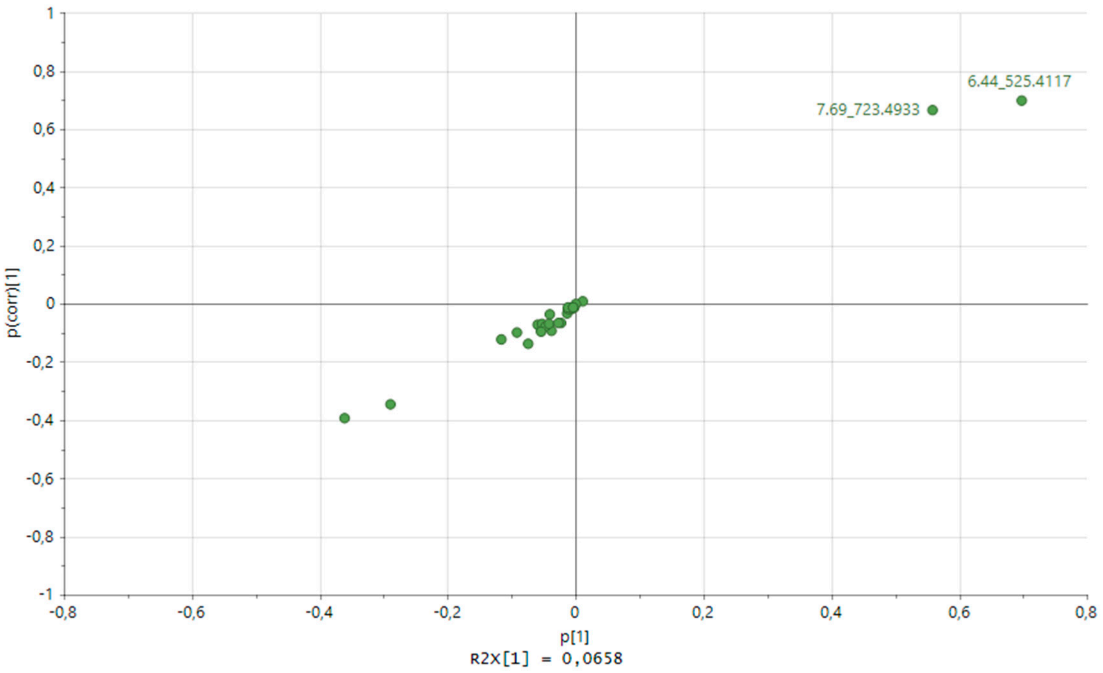

Figure S2: S-plot for fraction C, highlighting 2 potential biomarkers from fraction C with regards to Zf-NR bioactivity.
